# Preventing Perioperative Hypothermia in Neonatal Surgical Patients: A Phased Quality Improvement Initiative within the Wake Up Safe Collaborative

**DOI:** 10.1097/pq9.0000000000000895

**Published:** 2026-07-28

**Authors:** Lance S. Patak, Imelda M. Tjia, Thomas L. Shaw, Lynne Bedre

**Affiliations:** From the * Department of Anesthesiology and Pain Medicine, Cincinnati Children’s Hospital, Cincinnati, Ohio **the Department of Anesthesiology and Pain Medicine, Texas Children’s Hospital, Houston, Tex.; † Department of Anesthesiology and Pain Medicine, Texas Children’s Hospital, Houston, Tex.; ‡ Department of Nursing, Texas Children’s Hospital, Austin, Tex.

## Abstract

**Background:**

Neonates are at high risk for perioperative hypothermia, which is associated with increased morbidity and mortality. A serious safety event involving profound postoperative hypothermia prompted a quality improvement initiative to prevent perioperative hypothermia in neonatal intensive care unit surgical patients.

**Problem:**

A review of prior cases at our institution demonstrated that only 50% of neonates returned from surgery normothermic.

**Methods:**

A three-phase intervention was implemented: (1) a multidisciplinary hypothermia prevention checklist with standardized warming and transport processes; (2) assignment of a dedicated intraoperative “temperature guardian” responsible for continuous monitoring and team communication; and (3) implementation of dual-source intraoperative temperature monitoring. We collected perioperative temperature data across 39 consecutive surgical events.

**Interventions:**

Focused on standardizing preparation, clarifying team roles, enhancing intraoperative vigilance, and improving temperature measurement accuracy.

**Results:**

Implementation of phased interventions was associated with a reduction in hypothermia from 50% at baseline to 0% in subsequent phases, with elimination of hypothermia events and low rates of mild hyperthermia (≤20%), with 2 outlier events: 1 hypothermia emergency bedside procedure and 1 hyperthermia event with a falsely low core temperature reading during bowel irrigation. Checklist compliance improved to 92%. Intraoperative temperature monitoring compliance reached 100%.

**Conclusions:**

A structured, phased quality improvement approach emphasizing team accountability, standardized workflows, and validated monitoring reliably maintained perioperative normothermia in high-risk neonatal patients and offers a reproducible model for improving neonatal surgical safety.

## INTRODUCTION

### Problem Description

Perioperative hypothermia remains a common and preventable complication among neonates undergoing surgery. Neonates are particularly susceptible to rapid heat loss during anesthesia, surgical exposure, and transport due to immature thermoregulation, a high surface area-to-weight ratio, and minimal insulating fat.^1^ Even brief hypothermia episodes increase the risk of metabolic acidosis, coagulopathy, impaired wound healing, and mortality.^2–4^ Despite these known risks, achieving perioperative normothermia remains inconsistent, with some centers reporting compliance as low as 50%.^5^

At our institution, a serious safety event in which a neonate returned from surgery with an undetectable temperature highlighted a critical system vulnerability in perioperative thermoregulation. Similar to Cronin et al’s findings,^5^ subsequent review demonstrated inconsistent maintenance of normothermia, with only 50% of NICU surgical patients returning from the operating room (OR) within the normothermia range.

### Available Knowledge

Prior studies have demonstrated that bundled interventions such as prewarming, standardized checklists, environmental temperature control, and continuous intraoperative monitoring can reduce neonatal hypothermia in delivery rooms and perioperative settings.^6–8^ For example, targeted quality improvement interventions have successfully maintained normothermia during transport from the neonatal intensive care unit (NICU) to the OR, highlighting the importance of coordinated, multidisciplinary workflows.^8^ In pediatric perioperative care, modifiable perioperative factors such as OR temperature, use of warming devices, and continuous monitoring have also been identified as key determinants of thermal outcomes.^9^

Environmental conditions introduce additional complexity. OR temperature affects not only patient thermoregulation but also clinical and cognitive task performance, requiring a careful balance between patient safety and provider function.^10^ Despite these advances, maintaining consistent normothermia across all peri-anesthesia phases—including transport and intraoperative care—remains challenging. Prior studies have largely emphasized protocol standardization but have not consistently addressed intraoperative accountability, real-time vigilance during periods of high cognitive load, or validation of temperature measurements—factors that may contribute to persistent variability in outcomes.

**Fig. 1.**
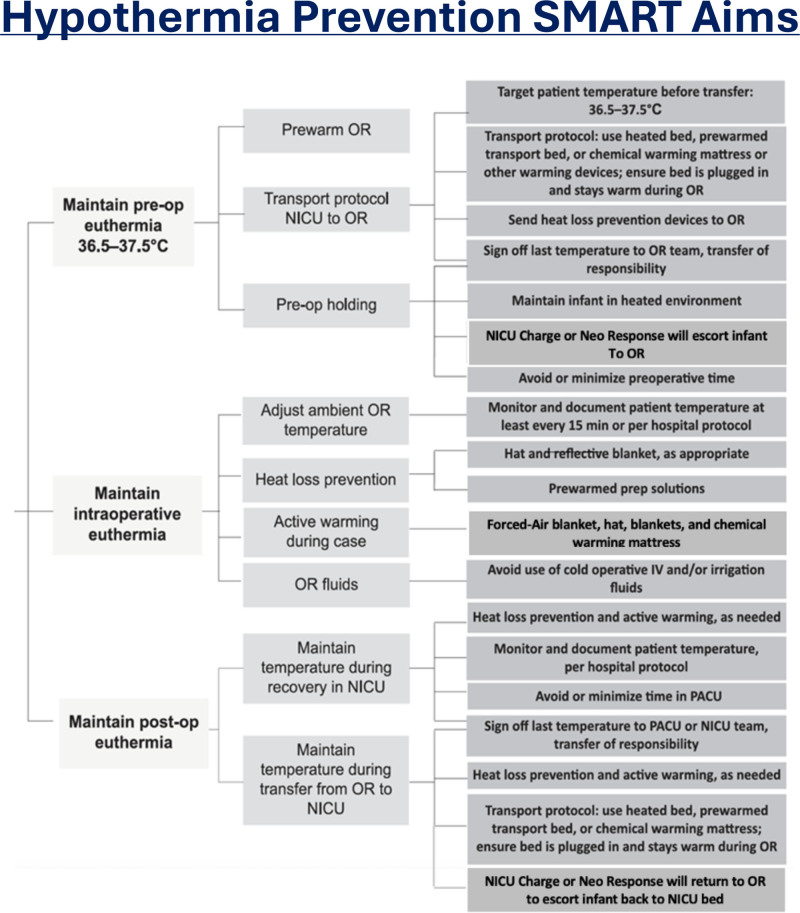
Key driver diagram for neonatal perioperative thermoregulation. The key driver diagram aligns with the SMART aim of reducing perioperative hypothermia (core temperature <36.5°C) in neonatal surgical patients. Primary drivers include standardized preoperative preparation, intraoperative vigilance, environmental temperature control, and reliable temperature monitoring. Secondary drivers and associated interventions are displayed.

The STEPP IN (Working Together to Keep Infants Warm in the Perioperative Period) collaborative, described by Brozanski et al, provides a conceptual framework for addressing these challenges by identifying key drivers of perioperative thermoregulation in neonates.^11^ We adapted this framework to develop a local key driver diagram, which informed the essential elements of our hypothermia prevention checklist and guided the selection of standardized process measures (Fig. 1).

### Rationale

Quality improvement theory emphasizes that complex patient safety problems are best addressed through system-level solutions rather than reliance on individual vigilance alone. Standardization, redundancy, and clearly defined roles are foundational principles for reducing variability and preventing harm.^12–16^ Environmental controls, such as OR temperature, represent system-level design decisions that influence both patient outcomes and clinician cognitive and task performance, underscoring the need for standardized, team-based approaches rather than individual adaptation.^10^

Participation in the Wake Up Safe (WUS) collaborative, a federally listed patient safety organization, provided a structured framework for shared learning and iterative testing of evidence-based interventions across institutions. Within this collaborative, a Neonatal Hypothermia Prevention Task Force, composed of nine member institutions, led a nationwide initiative to identify best practices for preventing perioperative hypothermia, a high-risk, high-impact safety concern. To address gaps in intraoperative accountability, real-time vigilance during periods of high cognitive load, and validation of temperature measurements, we designed a phased, system-based intervention that combined standardized workflows with novel strategies to enhance intraoperative reliability, including the introduction of a dedicated “temperature guardian” role and implementation of dual-source temperature monitoring to support real-time vigilance and measurement validation.

### Specific Aims

The primary aim of this quality improvement (QI) initiative was to reduce perioperative hypothermia (core temperature <36.5 °C) among NICU-transferred neonates undergoing surgery by implementing standardized, team-based thermoregulation interventions. A secondary aim was to identify key process changes associated with sustained maintenance of normothermia.

## METHODS

### Context

We conducted this initiative in the NICU of a tertiary pediatric hospital that participates in the WUS collaborative. We included all neonates undergoing surgical procedures either in the OR or at the bedside. This initiative followed a serious patient safety event in which a neonate returned from surgery and arrived at the NICU with an undetectable temperature. We assessed baseline performance through a retrospective review of neonatal surgical cases before implementing hypothermia prevention interventions. We defined normothermia as a documented temperature between 36.5 °C and 37.5 °C on return to the NICU. Clinical teams used variable temperature measurement methods in perioperative practice during the baseline period; however, NICU temperature measurements were consistent and axillary.

### Temperature Monitoring

We collected the following temperature data for all OR cases: (1) preoperative in NICU, (2) first temperature in the OR, (3) lowest OR temperature, (4) temperature upon leaving the OR, and (5) temperature upon return to NICU. Core temperature was monitored using standard institutional practices, which varied by case (eg, rectal, esophageal, or nasopharyngeal), and was not strictly controlled for this initiative. Four bedside procedures lacked intraoperative documented temperature values in the electronic medical record. In these cases, temperature monitoring was performed using the infant warmer; however, measurements were not automatically captured in the medical record and had to be entered manually. These procedures occurred under emergent conditions, during which manual temperature documentation was omitted. This finding indicated a lack of temperature monitoring and was addressed through subsequent education and process standardization.

### Study Population and Case Inclusion

We included all neonatal surgical and procedural cases requiring anesthesia support during the study period in the analysis, regardless of procedure location (OR or bedside) or urgency. We did not exclude emergent cases to reflect real-world clinical practice. For all cases, we reviewed preoperative and postoperative temperatures on return to the NICU to contextualize perioperative thermoregulatory performance, recognizing that baseline thermal status could influence postoperative outcomes, particularly in emergent bedside procedures.

### Interventions

We introduced interventions sequentially using iterative Plan-Do-Study-Act (PDSA) cycles.

#### Phase 1: Multidisciplinary Checklist and Data Tracking

**Fig. 2.**
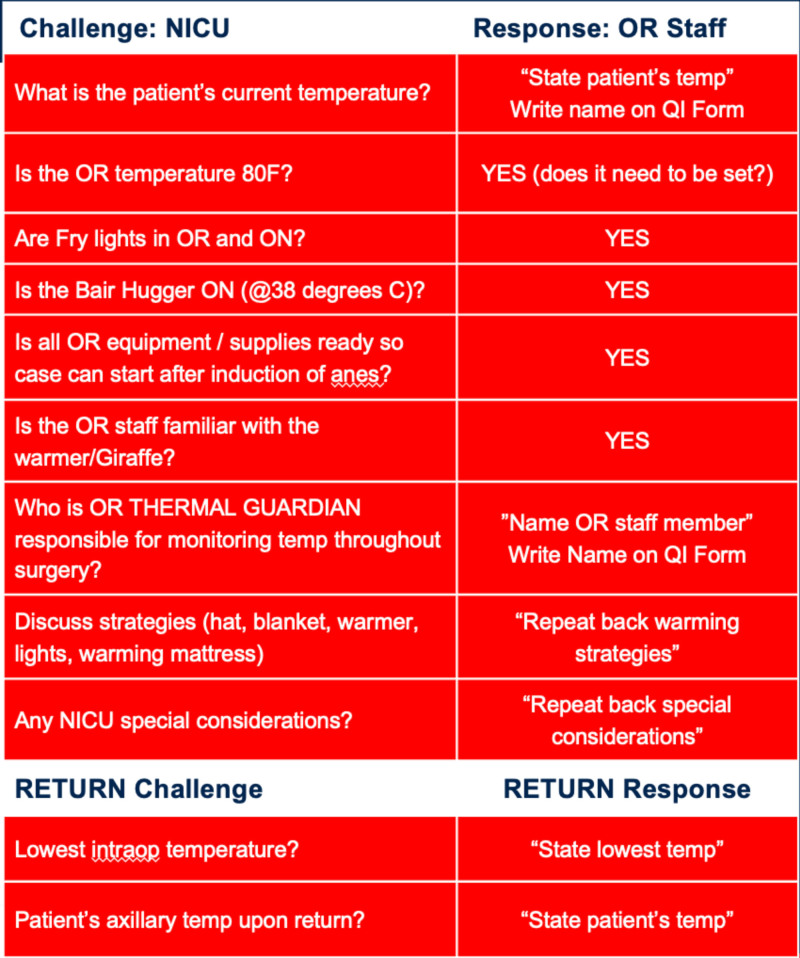
Multidisciplinary perioperative hypothermia prevention checklist. A standardized checklist used across NICU and OR teams to ensure consistent preparation, warming, and transport practices.

On September 2, 2024, a multidisciplinary perioperative hypothermia prevention checklist was introduced and first used on September 9, 2024, to share accountability among preoperative nursing, anesthesia, and NICU teams, consistent with prior QI studies showing that structured, system-level interventions and clearly defined responsibilities reduce perioperative hypothermia (Fig. 2 and **Supplemental Digital Content 1**, https://links.lww.com/PQ9/A773).^15,17^ We then standardized the perioperative hypothermia prevention checklist for all procedures to ensure consistent preparation, warming, and transport practices and tailored the accompanying QI data tracking forms to the procedure location, with separate forms for OR and bedside procedures to capture relevant process measures specific to each setting. The checklist was embedded in the preoperative workflow and required affirmative confirmation before proceeding with surgery in nonemergent cases; however, we did not independently audit adherence to individual checklist elements. Key elements of the checklist and tracking forms included:

- Setting OR temperature to 80 °F (26.7 °C) before patient arrival.
- Avoiding surgical start delays and unnecessary neonatal exposure by ensuring preparation of all supplies in advance of patient arrival.
- Ensuring availability of a designated, trained preoperative team member to manage the neonatal warmer (eg, perioperative nurse or NICU nurse).
- Using warming lights during periods of exposure.
- Transporting the patient from the OR in a prewarmed bed, irrespective of bed type (eg, Giraffe OmniBed Carestation, GE Healthcare, Chicago).

#### Phase 2: Intraoperative Temperature Guardian

We assigned the OR circulating nurse to serve as the intraoperative “temperature guardian” to address lapses in intraoperative monitoring during periods of high cognitive load (eg, airway management). This team member was responsible for:

- Initiating temperature monitoring immediately upon OR arrival.
- Continuously surveilling temperature trends and alerting the team when core temperature approached predefined thresholds (36.5 °C and 37.5 °C).

#### Phase 3: Dual-source Temperature Monitoring

We implemented dual-source temperature monitoring after identifying a hyperthermia event in which bowel irrigation caused the rectal core temperature to read falsely low, prompting inappropriate warming measures. Because the two temperature sources were expected to differ in absolute value, their combined use provided redundancy, enabling validation of unexpected readings and preventing inappropriate warming or cooling interventions. The purpose of dual monitoring was to assess directional trends over time rather than rely on a single absolute value; discordant trends prompted clinical investigation for potential causes such as probe displacement, environmental exposure, or irrigation effects, rather than automatic warming or cooling interventions.

### Study of the Interventions

We compared perioperative temperature outcomes over time and identified deviations associated with process gaps or system stressors (eg, prolonged airway management, lengthy surgical preparation) to assess the impact of our interventions.

### Measures

The NICU nursing staff measured axillary postoperative temperatures immediately upon return to the NICU. The primary outcome measure was temperature on return to the NICU, categorized as hypothermia (<36.5 °C), normothermia (36.5–37.5 °C), or hyperthermia (>37.5 °C). Secondary process measures included confirmation of checklist activation and timing of temperature monitoring initiation. We obtained data from QI tracking forms and verified them against the electronic medical record.

### Analysis

Descriptive statistics and statistical process control charts evaluated trends over time, consistent with quality improvement methodology for detecting nonrandom variation in small samples.^18^ We applied standard rules for detecting special cause variation, including shifts and trends. A scatter plot was used to explore the association between patient weight and postoperative temperature. Learning from outliers informed subsequent PDSA cycles.

### Ethical Considerations

Operating under a multisite institutional review board, participating institutions within the WUS patient safety organization framework contribute de-identified adverse event data to a centralized registry, enabling identification of system-level contributors to harm and dissemination of best practices. Each participating site implements targeted interventions tailored to its local environment and contributes data toward a shared goal of maintaining normothermia during high-risk peri-anesthesia periods, including transport to and from the NICU. The work described here represents one institution’s experience within this multicenter initiative, highlighting site-specific interventions, outcomes, and lessons learned to inform the consortium’s broader effort to establish best practices for neonatal thermoregulation and surgical safety.

## RESULTS

### Participants and Case Distribution

**Fig. 3.**
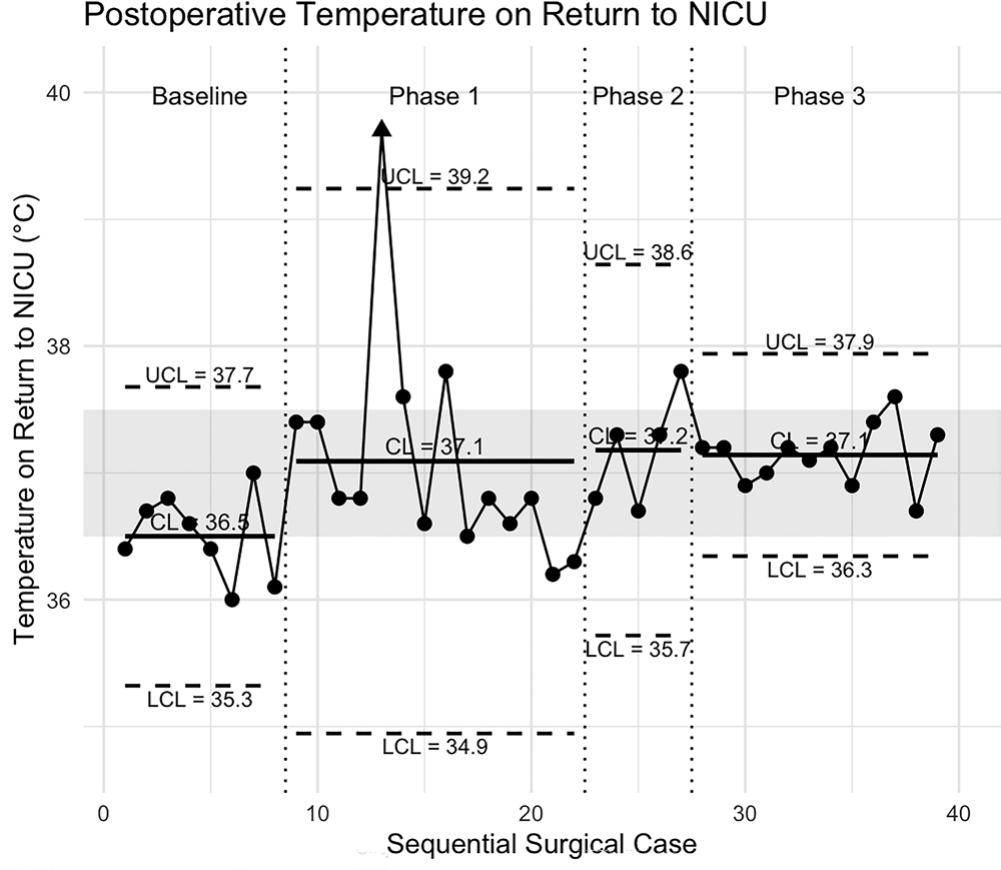
Postoperative temperature on return to the NICU across sequential hypothermia prevention phases. An individual chart displaying postoperative axillary temperature on return to the NICU for 39 sequential surgical cases. Solid horizontal lines represent phase-specific mean temperatures. Dashed lines indicate upper and lower control limits, calculated separately within each phase, based on point-to-point variability using the moving-range method. The shaded band indicates the clinical normothermia target range (36.5–37.5°C). Vertical dotted lines mark transitions between the baseline period and successive intervention phases. Phase-specific estimates should be interpreted with caution due to the limited number of observations in some phases.

We collected perioperative temperature data from 39 consecutive surgical events, including 8 preintervention cases (**Supplemental Digital Content 2**, https://links.lww.com/PQ9/A774). The cases were distributed across the 3 QI phases as follows: phase 1 (checklist only): 14 cases; phase 2 (temperature guardian): 5 cases; phase 3 (dual-source monitoring): 12 cases (Fig. 3). Bedside procedures were disproportionately represented among hypothermia events and were typically associated with emergent conditions and limited preoperative optimization.

### Baseline Measurement

During the preintervention period, only 8 neonatal surgical cases occurred over a 3-month interval, reflecting the hospital’s early operational phase (**Supplemental Digital Content 2**, https://links.lww.com/PQ9/A774). Of these, 4 returned to the NICU within the normothermic range, corresponding to a baseline normothermic rate of approximately 50%, highlighting substantial variability in perioperative temperature management before standardized interventions.

### Incidence of Hypothermia and Hyperthermia by Phase

Postoperative temperature outcomes by intervention phase are summarized in Table 1. During phase 1 (checklist implementation), 2 of 14 patients (14%) were hypothermic on return to the NICU, including 1 bedside procedure, and 3 patients (21%) were mildly hyperthermic. The hypothermic bedside case occurred in a patient who was hypothermic before anesthesia induction. Following the implementation of the intraoperative temperature guardian in phase 2, no hypothermia events occurred among 5 patients, and 1 mild hyperthermia event (20%) occurred. Similarly, during phase 3, which included dual-source temperature monitoring, no hypothermia events were observed among 12 patients, and 1 mild hyperthermia event (8%) was associated with a falsely low core temperature reading.

**Table 1.** Incidence of Hypothermia and Hyperthermia by Phase

Phase	Cases, n	Hypothermia, n (%)	Hyperthermia, n (%)	Notes/Outliers
1. Checklist	14	2 (14)	3 (21)	Hypothermia during bedside procedure
2. Temperature guardian	5	0 (0)	1 (20)	Mild hyperthermia
3. Dual-source monitoring	12	0 (0)	1 (8)	Hyperthermia due to false-low core reading

### Special Cause Variation/Outliers

We identified 2 events as special cause variation:

1. A hypothermic neonate during an emergent bedside procedure was hypothermic before surgery due to limited preoperative warming opportunities.
2. A hyperthermic neonate after bowel irrigation, where the rectal core temperature read falsely low, led to escalated warming measures until the error was recognized.

### Compliance

- Checklist completion improved from ~75% initially to 92% after process refinement (assignment of responsibility to the NICU charge nurse).
- QI Data Form collection was frequently incomplete, requiring audit review of 100% of cases to both verify and complete temperature recordings.
- Intraoperative temperature monitoring compliance, including the presence of the temperature guardian and dual-source monitoring, reached 100% in phases 2 and 3.

### Countermeasures

- Staff retraining and refresher education on checklist use and infant warmer management.
- Adjustments to warming protocols, including prewarmed transport beds and use of warming lights during exposure.
- Implementation of dual-source temperature monitoring to verify anomalous readings and prevent inappropriate warming or cooling.
- Debriefing sessions after outlier events to identify process vulnerabilities and implement corrective strategies.

**Fig. 4.**
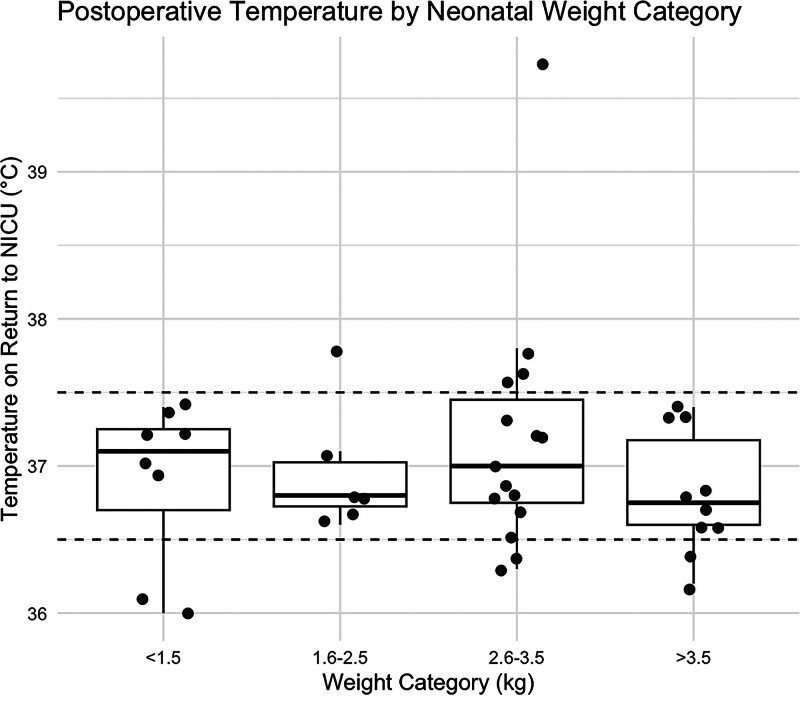
Postoperative temperature by neonatal weight category. The distribution of axillary temperatures on return to the NICU across predefined neonatal weight categories. Each point represents an individual case (n = 39). Dashed lines indicate the normothermic range (36.5–37.5°C). No meaningful differences in temperature distribution were observed across weight groups.

The analysis showed no meaningful correlation between body weight and postoperative temperature, suggesting that process reliability and intraoperative monitoring practices were more influential than patient size alone (Fig. 4). Deviations from the target temperature range were treated as learning opportunities, providing insights for process refinement rather than representing system failures. These findings informed targeted countermeasures, including reinforcement of checklist use, staff education, and implementation of dual-source temperature monitoring to rapidly detect and correct anomalous readings, thereby ensuring consistent maintenance of normothermia.

### Process Measures and Implementation Fidelity

Use of a checklist was required before surgery for all nonemergent cases. Although formal element-by-element compliance auditing was not performed, checklist completion was confirmed in all scheduled OR cases through preoperative workflow requirements. Completion of accompanying QI data-tracking forms was inconsistent throughout the initiative, prompting reliance on electronic medical record review to verify all temperature outcomes and limiting the temperature data for analysis to only those values auto-captured or documented by NICU nursing staff. We structured ongoing education as a combination of onboarding training, periodic unit-based reinforcement, safety huddles, and targeted just-in-time education informed by outcome reviews, rather than fixed-interval retraining.

### Summary

After implementing these measures, normothermia was consistently maintained across all subsequent surgical cases, with only 2 mild hyperthermia events, demonstrating the interventions’ effectiveness and sustainability. Continuous monitoring, clear role accountability, and system-level redundancy were key to success.

## DISCUSSION

### Summary

This QI initiative achieved sustained prevention of perioperative hypothermia in neonatal surgical patients through a phased, system-based approach. The intraoperative temperature guardian was strongly associated with the maintenance of normothermia. Overall, the most effective interventions were focused warmer education and a dedicated intraoperative temperature guardian, emphasizing clearly defined roles and continuous monitoring in perioperative thermoregulation.^15,16^ These findings are consistent with prior work demonstrating that structured QI interventions can significantly reduce intraoperative hypothermia in both NICU infants and broader pediatric populations.^15,16^ Although multiple interventions were implemented, the elimination of hypothermia events followed the introduction of focused warmer education and the temperature guardian, suggesting these were key drivers of improved temperature stability. These interventions improved reliability by addressing distinct sources of error. The checklist reduced omission errors by standardizing preparation; the temperature guardian mitigated attentional lapses under high cognitive load; and dual-source monitoring reduced measurement error by enabling validation of unexpected values. Together, these complementary strategies improved overall system reliability.

### Interpretation

Early challenges with checklist adoption among anesthesia personnel underscored the importance of engaging NICU leaders to take ownership of this process and ensure role clarity and workflow integration. Relocating checklist initiation to the NICU and assigning responsibility to the charge nurse improved reliability. A designated temperature guardian and redundant monitoring supported intraoperative vigilance during periods of high cognitive load. We supported sustainability by embedding the checklist into routine workflow, assigning the temperature guardian role on a per-case basis rather than to specific individuals, and incorporating thermoregulation education into staff onboarding and ongoing safety reinforcement. We also established a NICU hypothermia prevention committee to review outcomes and support ongoing improvement. This approach aligns with improvement science frameworks emphasizing learning systems and sustained improvement through embedded workflows.^19^

### Comparison with Prior Studies and Novel Contributions

Several prior quality improvement initiatives published in *Pediatric Quality & Safety* have demonstrated that bundled interventions—such as checklists, prewarming protocols, and environmental controls—can reduce perioperative hypothermia in neonatal and pediatric populations.^8,9,17^ Our work aligns with these studies in demonstrating that structured, multidisciplinary interventions improve thermoregulation outcomes. However, our approach differs in several important ways.

First, whereas prior studies typically implemented bundled interventions simultaneously, we introduced the interventions sequentially, allowing us to evaluate the incremental impact of individual process changes. This phased approach enabled targeted assessment and refinement, and the introduction of a dedicated intraoperative temperature guardian was strongly associated with the elimination of hypothermia events.

Second, most prior studies emphasize protocol standardization, whereas our findings highlight the importance of real-time intraoperative accountability during periods of high cognitive load. The temperature guardian directly addressed attentional lapses during critical intraoperative tasks.

Third, dual-source temperature monitoring represents a novel contribution that addresses measurement reliability, an area that has been less emphasized in prior QI work. By identifying falsely low readings, this intervention prevented inappropriate warming and reduced the risk of hyperthermia.

Finally, our study extends prior work by incorporating detailed failure analyses and outlier evaluation, providing practical insights into system vulnerabilities, such as bedside procedures, prolonged induction, and (false-low) core temperature readings. Together, these findings suggest that combining standardization with active surveillance and measurement validation may enhance reliability beyond traditional bundled approaches. Conducted within the WUS collaborative, this work also demonstrates how local interventions can align with a multicenter safety framework to support shared learning and reproducibility.

### Limitations

This single-center initiative may have limited generalizability to institutions with different resources or workflows. The sample size was modest, and data collection relied in part on manually completed QI forms, introducing potential reporting bias. We did not control for confounding variables such as procedure type and duration. The specific site of core temperature measurement varied by patient, procedure, and anesthesia provider. Although this variability may affect absolute values, the interventions focused on maintaining consistent perioperative normothermia across cases rather than a specific measurement site. Nonetheless, outcome data were verified against the electronic medical record, and results were consistent over time.

Only 4 procedures in this cohort were performed at the bedside rather than in the OR. Among these cases, 2 patients were normothermic, and 2 were hypothermic upon completion of each case. Given the small number of bedside procedures, a meaningful comparison between bedside and OR cases was not possible. In addition, bedside procedures were typically emergent, with limited opportunity to optimize preoperative thermal status. As a result, perioperative temperatures in these cases likely reflected the patient’s baseline condition and clinical urgency rather than the effectiveness of intraoperative thermoregulation alone.

Although we monitored process reliability through checklist activation, role assignment, and audit review, detailed element-level compliance data were not consistently available. The hospital’s early operational phase may also have contributed to variability in documentation and learning-curve effects. We cannot exclude secular trends or increased team awareness over time as contributors to improved outcomes.

## CONCLUSIONS

A structured, phased quality improvement initiative emphasizing standardized processes, clearly defined roles, and continuous, validated monitoring was associated with the elimination of perioperative hypothermia events in high-risk neonates. Implementing a multidisciplinary checklist, a dedicated intraoperative temperature guardian, and dual-source monitoring offers a practical, reproducible approach for improving neonatal surgical safety and may inform multicenter efforts to establish best practices in perioperative thermoregulation.

## ETHICAL APPROVAL

This study was conducted with approval from the IRB H-20107.

## Supplementary Material

## References

[1] Vinci A Islam S Quintos-Alegheband L. A quality improvement intervention to decrease hypothermia in the delivery room using a checklist. Pediatr Qual Saf. 2018;3:e125. 31334457 10.1097/pq9.0000000000000125 PMC6581478

[2] Zeiner S Zadrazil M Willschke H. Accuracy of a dual-sensor heat-flux (DHF) non-invasive core temperature sensor in pediatric patients undergoing surgery. J Clin Med. 2023;12:7018. 38002632 10.3390/jcm12227018 PMC10672443

[3] Baracetti M Hagos E Tolera J. Effectiveness of bundled interventions for the prevention of neonatal hypothermia in low-income settings: a quality improvement project in a referral hospital in Ethiopia. Children (Basel). 2025;12:709. 40564667 10.3390/children12060709 PMC12191039

[4] Choi HS Lee SM Eun H. The impact of a quality improvement effort in reducing admission hypothermia in preterm infants following delivery. Korean J Pediatr. 2018;61:239–244. 30130949 10.3345/kjp.2018.61.8.239 PMC6107400

[5] Cronin JA Soghier L Ryan K. A quality initiative for reducing postoperative hypothermia for neonatal intensive care unit surgical patients. Pediatr Qual Saf. 2020;5:e318. 32766492 10.1097/pq9.0000000000000318 PMC7360329

[6] Hanna M Htun Z Islam S. A quality improvement initiative to improve perioperative hypothermia rates in the NICU utilizing checklists. Pediatr Qual Saf. 2020;5:e367. 33062906 10.1097/pq9.0000000000000367 PMC7470004

[7] Engorn BM Kahntroff SL Frank KM. Perioperative hypothermia in neonatal intensive care unit patients: effectiveness of a thermoregulation intervention and associated risk factors. Paediatr Anaesth. 2017;27:196–204. 27917566 10.1111/pan.13047

[8] Studer A Fleming B Jones RC. Reducing intraoperative hypothermia in infants from the neonatal intensive care unit. Pediatr Qual Saf. 2023;8:e655. 10.1097/pq9.0000000000000665 PMC10332830 37434591

[9] Lee SY Wan SYK Tay CL. Perioperative temperature management in children: what matters? Pediatr Qual Saf. 2020;5:e350. 34616966 10.1097/pq9.0000000000000350 PMC8487781

[10] Hakim M Walia H Dellinger HL. The effect of operating room temperature on the performance of clinical and cognitive tasks. Pediatr Qual Saf. 2018;3:e069. 30280125 10.1097/pq9.0000000000000069 PMC6132757

[11] Brozanski BS Piazza AJ Chuo J. STEPP IN: working together to keep infants warm in the perioperative period. Pediatrics. 2020;145:e20191121. 32193210 10.1542/peds.2019-1121

[12] Reason J. Human error: models and management. BMJ. 2000;320:768–770. 10720363 10.1136/bmj.320.7237.768 PMC1117770

[13] Madine M Simsekler MCE Salah K. Applying quality improvement science to patient safety: strategies, frameworks, and sustainable solutions. Risk Manag Healthc Policy. 2025;18:3781–3791. 41367428 10.2147/RMHP.S564459 PMC12684984

[14] Pichumani A Engel C Dhingra N. Safe Care is the Right Care: ISQua White Paper on Patient Safety for Healthcare Organisations. International Society for Quality in Health Care; 2025. https://isqua.org/media/attachments/2025/04/02/isqua-white-paper-on-patient-safety-in-healthcare-organisations.pdf. Accessed January 30, 2026. 10.1093/intqhc/mzaf071 40795043

[15] Fricke J Galligan M Douma C Souder J Hedden-Gross A Mull N. Examining the Impact of Implementing High-Reliability Organization Principles on Patient Safety Outcomes: Rapid Review. 2025 May. In: Making Healthcare Safer IV: A Continuous Updating of Patient Safety Harms and Practices. Rockville (MD): Agency for Healthcare Research and Quality (US); 2023. 41248244

[16] Shi Y Miao S Fu Y. TeamSTEPPS improves patient safety. BMJ Open Qual. 2024;13:e002669. 10.1136/bmjoq-2023-002669 PMC11057264 38670556

[17] Wong BJ Rama A Caruso TJ. A pilot quality improvement project to reduce intraoperative MRI hypothermia in neurosurgical patients. Pediatr Qual Saf. 2022;7:e531. 35369418 10.1097/pq9.0000000000000531 PMC8970077

[18] Perla RJ Provost LP Murray SK. The run chart: a simple analytical tool for learning from variation in healthcare processes. BMJ Qual Saf. 2011;20:46–51. 10.1136/bmjqs.2009.037895 21228075

[19] Dixon-Woods M. How to improve healthcare improvement-an essay by Mary Dixon-Woods. BMJ. 2019;367:l5514. 31575526 10.1136/bmj.l5514 PMC6768008

