# Supplementary material for: Preventing Perioperative Hypothermia in Neonatal Surgical Patients: A Phased Quality Improvement Initiative within the Wake Up Safe Collaborative
Source: Pediatr Qual Saf. 2026 Jul 28;11(4):e895. doi: 10.1097/pq9.0000000000000895 (PMC13412708; doi:10.1097/pq9.0000000000000895)
Supplement: Supplementary file 1 [file pqs-11-e895-s001.pdf]

Supplemental Digital Content 1. QI Data Tracking Forms

TCH NICU Bedside Surgery Thermal Defense Plan

(Version 3/5/2025)

NICU Bedside Surgery

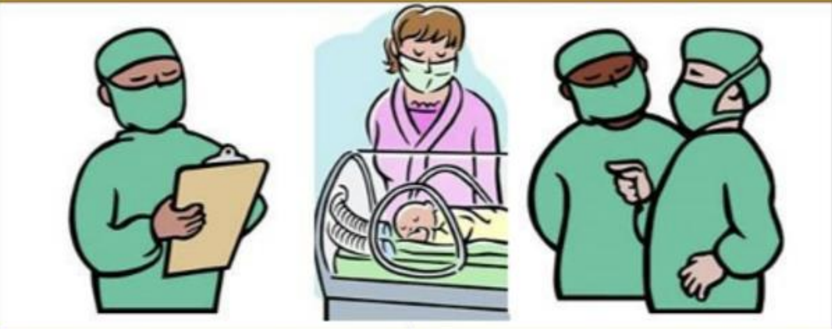

Preoperative AX TEMP	Postoperative AX TEMP
____________°C	____________°C

- ❑ Radiant Warmer or Giraffe Omnibed (if < 1.5kg) on Servo Control
- ❑ Transwarmer Mattress (Needs to be Changed Q30")
- ❑ Warm Blankets
- ❑ Hat
- ❑ Bair Hugger
- ❑ Heated Breathing Circuit
- ❑ Esophageal Temp Probe

Team will be monitoring Temperature by:

- ❑ Axillary Temperature (Range: 97.7-99.6°F)
- ❑ Esophageal Temperature Probe (Range: 36.5-37.5°C)
- ❑ Skin Temperature Probe (Range: 36.5-37.5°C)
- ❑ Temperature Guardian ______________________

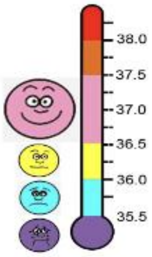

Date:___________

Patient Sticker

QUALITY DATA: NOT PART OF THE PERMANENT MEDICAL RECORD.
This information is privileged and confidential pursuant to Texas Health & Safety Code sections 161.031-161.033 and Texas Occupations Code section 160.007 and/or T.R.C.P. 192.5

Texas Children's Hospital OR Thermal Defense Plan

(Version 3/5/2025)

NICU RN	OR RN	OR RN	NICU RN
NICU to OR<br>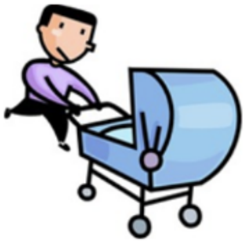	Room Arrival<br>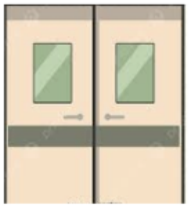	Procedure Start<br>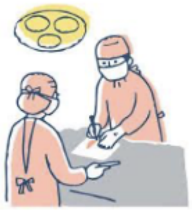	OR to NICU
	Intraop Thermal Guardian:<br>*Team member who will be monitoring temp during induction/skin prep<br>________________________		
**NICU PreOp AX TEMP**<br>__________°F	**OR Arrival Temp**<br>__________°F	**Procedure Start Temp**<br>__________°F	**NICU PostOp AXTEMP**<br>__________°F
❑ Hat<br>❑ TransWarmer Gel Mattress<br>❑ Warm Blankets<br>❑ Warmer (or)<br>❑ Giraffe isolette for infants<1.5Kg*	❑ Increase Room temp to 80°F<br>❑ Setup NICU radiant warmer (fry lights)<br>❑ Setup Mistral Air (bair hugger)	❑ Mistral Air (bair hugger)<br>❑ Webril available (for extremity wraps)<br>❑ Heated Circuit<br>❑ Fluid Warmer for irrigation	❑ Hat<br>❑ TransWarmer Gel Mattress<br>❑ Warm Blankets

*A NICU RN will escort OR team to assist with the Giraffe Isolette

Axillary/Esophageal/Skin Temp Range:

36.5-37.5°C

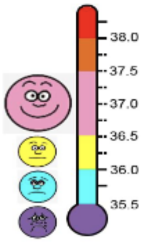

*Please call ATX NICU Charge RN if you have questions/issues: 737-229-2920

Return form to NICU Charge Nurse or Anesthesia QI Box

Date:___________

Patient Sticker

QUALITY DATA: NOT PART OF THE PERMANENT MEDICAL RECORD.
This information is privileged and confidential pursuant to Texas Health & Safety Code sections 161.031-161.033 and Texas Occupations Code section 160.007 and/or T.R.C.P. 192.5
