# Supplementary material for: Preventing Perioperative Hypothermia in Neonatal Surgical Patients: A Phased Quality Improvement Initiative within the Wake Up Safe Collaborative
Source: Pediatr Qual Saf. 2026 Jul 28;11(4):e895. doi: 10.1097/pq9.0000000000000895 (PMC13412708; doi:10.1097/pq9.0000000000000895)
Supplement: Supplementary file 2 [file pqs-11-e895-s002.pdf]

Supplemental Digital Content 2. Perioperative Temperature Data by Surgical Case Across Sequential Hypothermia Prevention Phases

Baseline Data

Surgical Event	Temperature on Return to NICU (°C)	Intraoperative Nadir Temperature (°C)	Weight (kg)
1	36.4	35.0	2.90
2	36.7	36.5	2.90
3	36.8	36.8	3.40
4	36.6	36.2	7.00
5	36.4	35.8	5.60
6	36.0	Not recorded (bedside procedure)*	0.62
7	37.0	Not recorded (bedside procedure)*	0.62
8	36.1	33.3	0.84

* Intraoperative nadir temperature was not recorded for select emergent bedside procedures due to lack of automated documentation capture.

Phase 1 – Implementation of OR Departure Checklist

Surgical Event	Temperature on Return to NICU (°C)	Intraoperative Nadir Temperature (°C)	Weight (kg)
9	37.4	36.9	3.60
10	37.4	36.8	1.40
11	36.8	35.5	2.90
12	36.8	35.2	3.60
13	39.7	36.3	3.10
14	37.6	37.4	3.30
15	36.6	36.2	1.60
16	37.8	36.9	2.60
17	36.5	35.3	2.60
18	36.8	36.4	2.20
19	36.6	Not recorded (bedside procedure)*	3.80
20	36.8	35.6	4.50
21	36.2	Not recorded (bedside procedure)*	3.80
22	36.3	33.5	3.50

* Intraoperative nadir temperature was not recorded for select emergent bedside procedures due to lack of automated documentation capture.

Phase 2 – Implementation of Intraoperative Temperature Guardian

Surgical Date	Temperature on Return to NICU (°C)	Intraoperative Nadir Temperature (°C)	Weight (kg)
23	36.8	37.0	2.30
24	37.3	35.3	5.30
25	36.7	36.4	4.20
26	37.3	36.3	2.98
27	37.8	35.7	2.15

Phase 3 – Implementation of Dual-Source Temperature Monitoring

Surgical Event	Temperature on Return to NICU (°C)	Intraoperative Nadir Temperature (°C)	Weight (kg)
28	37.2	36.6	2.96
29	37.2	35.9	3.43
30	36.9	36.4	2.89
31	37.0	35.9	3.03
32	37.2	36.2	0.92
33	37.1	35.1	2.00
34	37.2	36.4	0.92
35	36.9	37.1	0.92
36	37.4	35.8	0.92
37	37.6	35.7	2.60
38	36.7	35.6	1.54
39	37.3	36.0	4.40
